# Training through malaria research: building capacity in good clinical and laboratory practice in Liberia

**DOI:** 10.1186/s12936-019-2767-1

**Published:** 2019-04-17

**Authors:** Alfredo Mayor, Guillermo Martínez-Pérez, Christine K. Tarr-Attia, Bondey Breeze-Barry, Adelaida Sarukhan, Ana Meyer García-Sípido, Juan Carlos Hurtado, Dawoh Peter Lansana, Núria Casamitjana

**Affiliations:** 1 ISGlobal, Hospital Clínic, Universitat de Barcelona, Barcelona, Spain; 2 Saint Joseph’s Catholic Hospital, Monrovia, Liberia; 3 Juan Ciudad Foundation, Madrid, Spain

**Keywords:** Research capacity strengthening, Training, Good Clinical Laboratory Practice, Malaria, Pregnancy

## Abstract

**Background:**

Limited health research capacities (HRC) undermine a country’s ability to identify and adequately respond to local health needs. Although numerous interventions to strengthen HRC have been conducted in Africa, there is a need to share the lessons learnt by funding organizations, institutes and researchers. The aim of this report is to identify best practices in HRC strengthening by describing a training programme conducted between 2016 and 2017 at the Saint Joseph’s Catholic Hospital (SJCH) in Monrovia (Liberia).

**Methods:**

A call for trainees was launched at the SJCH, the Liberia Medicines and Health Products Regulatory Authority (LMHRA), the Ministry of Health and Social Welfare, the Mother Pattern College of Health Sciences (MPCHS) and community members. Selected trainees participated in four workshops on Good Clinical Laboratory Practice (GCLP), standard operating procedures (SOP) and scientific communication, as well as in a 5-months eLearning mentoring programme. After the training, a collectively-designed research project on malaria was conducted.

**Results:**

Twenty-one of the 28 trainees (14 from the SJCH, 3 from LMHRA, one from MPCHS, and 10 community representatives) completed the programme satisfactorily. Pre- and post-training questionnaires completed by 9 of the trainees showed a 14% increase in the percentage of correct answers. Trainees participated in a mixed-methods cross-sectional study of *Plasmodium falciparum* infection among pregnant women at the SJCH. Selected trainees disseminated activities and research outcomes in three international meetings and three scientific publications.

**Conclusion:**

This training-through-research programme successfully involved SJCH staff and community members in a practical research exercise on malaria during pregnancy. The challenge is to ensure that the SJCH remains active in research. Harmonization of effectiveness indicators for HRC initiatives would strengthen the case for investing in such efforts.

**Electronic supplementary material:**

The online version of this article (10.1186/s12936-019-2767-1) contains supplementary material, which is available to authorized users.

## Background

Health research promotes societal progress [1] by generating the knowledge needed to improve health systems performance and, ultimately, health and health equity [2, 3]. Strengthening health research capacities can enhance the ability of nations to improve their health outcomes [4]. Despite this awareness, research inequities persist in low- and middle-income countries (LMIC), with sub-Saharan Africa (SSA) experiencing one of the world’s most severe shortages in technical and human capacities, undermining their ability to respond to local health needs [4–7].

Efforts to increase individual and institutional ability to undertake high-quality research and to engage with community and stakeholders are key to strengthening health systems in the SSA region [8]. Despite substantial investment in research capacity strengthening [6, 7, 9–13], evaluations of the effectiveness of these programmes are scarce. Outcome indicators vary among initiatives, including citation analysis [14–16], measures of knowledge change pre- and post-intervention [17–20] and other types of ‘attributional’ measures that relate capacity improvement with the intervention [17, 20–22]. The lack of published real-life examples of capacity building and practical evaluation indicators [23] hinder the ability to determine whether resources invested in capacity building are being effective in achieving sustainability. Wider dissemination of specific cases is needed to draw lessons from capacity-building initiatives.

The mismatch between increased disease burden and research capacities in SSA has likely contributed to the region’s limited capacity to respond to epidemic outbreaks. A paradigmatic example is the 2014–2015 Ebola epidemic, the longest and deadliest in documented history, resulting in 28,616 cases and 11,310 deaths and the reversal of a decade of health and economic gains in Guinea, Liberia, and Sierra Leone. Though a pioneer country in tropical medicine research [24–27], Liberia was poorly prepared to support clinical research during the Ebola outbreak. The National Reference Laboratory in Charlesville was dismantled and the laboratory of the Liberian Institute for Biomedical Research (LIBR) remained entirely dedicated to run PCR tests for Ebola virus. As a result of the crisis, health services for diseases other than Ebola, such as malaria or measles, were severely disrupted [28, 29] causing an increase in morbidity and mortality, and decimating research and clinical capacities [30].

**Fig. 1:**
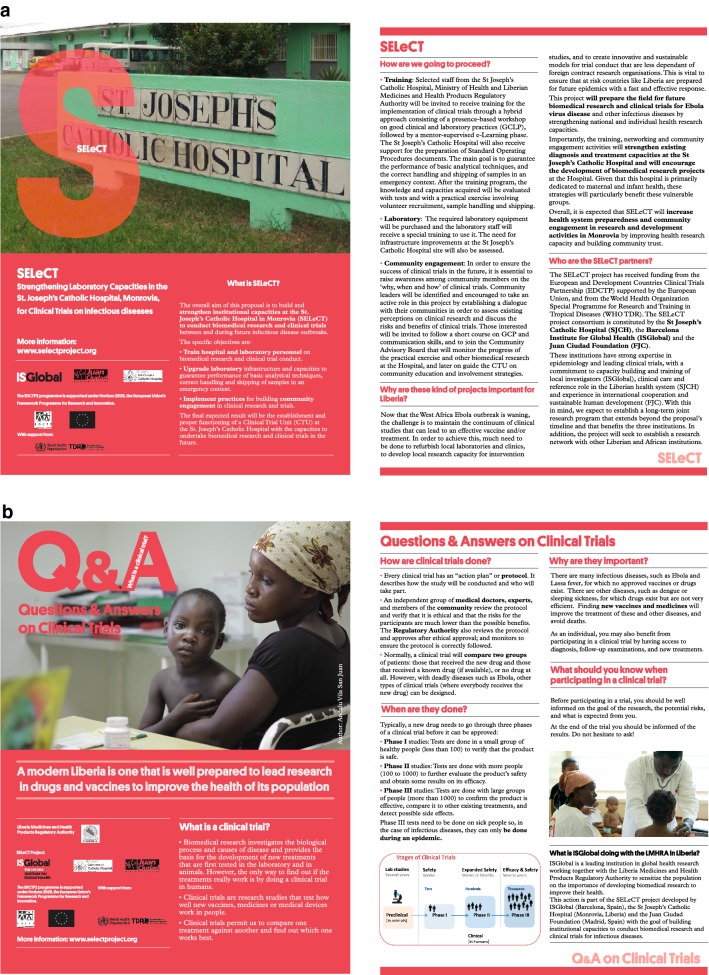
Brochure describing the objectives of the SELeCT project (**a**) and the value of clinical trials for biomedical research (**b**)

In this post-Ebola scenario, efforts to strengthen health system capacities to support epidemic preparedness and health research were urgently needed. With this in mind, the European and Developing Countries Clinical Trial Partnership (EDCTP) and the World Health Organization Special Programme for Research and Training in Tropical Diseases (WHO-TDR) launched in 2015 a call for initiatives to build and strengthen capacities to conduct high quality health research during infectious disease outbreaks. The capacity-building programme ‘*Strengthening laboratory capacities in the St. Joseph’s Catholic Hospital (SJCH), Monrovia, for clinical trials on infectious diseases*’ (SELeCT) was implemented to build and strengthen health research capacities at the SJCH in Monrovia (Liberia; Fig. 1). A training programme in Good Clinical and Laboratory Practice (GCLP) was proposed as a key intervention to help SELeCT achieve its aim. The training included a practical research exercise on malaria which was collectively designed, giving the trainees the opportunity to apply and strengthen the acquired capacities. The aim of this paper is to describe the methodology used during the training programme and the main outputs of its implementation, to identify key barriers to clinical research capacity building, and to provide insights on how to overcome these obstacles.

## Methods

### Period and organizations involved

The training programme on biomedical research was implemented between February 2016 and July 2017 by a consortium of three organizations: the Barcelona Institute for Global Health (ISGlobal, Spain) as coordinating institution, the non-governmental organization Juan Ciudad Foundation (JCF, Spain), and the Saint Joseph’s Catholic Hospital (SJCH, Liberia) where all training activities took place.

The SJCH is a secondary-level hospital located in Monrovia’s Congo Town neighborhood. The Hospital, opened in 1963, is run by the Brothers of the Hospitallier Order of St. John of God. It has a capacity of 142 beds, around 190 workers, and offers Internal Medicine, General Surgery, Obstetrics and Gynecology, Paediatrics and 24-h emergency services. It also participates in the National Treatment HIV (AIDS) and the Tuberculosis programmes. The SJCH closed its facilities between August and November 2014 due to the Ebola outbreak, which directly affected 13 workers, 9 of whom died, including the Hospital director, doctors, nurses and a laboratory technician. The Hospital reopened its maternity unit on November 24, 2014 and its paediatric service on February 2, 2015. At reopening, the activities implemented were outpatient screening, holding suspected cases (all cases presenting Ebola-like symptoms) at the Community Care Center, and inpatient care. In March 2015, approximately 550 rapid diagnostic tests (RDT) for malaria, 930 antenatal care visits and 360 paediatric visits at the outpatient clinics (296 under 5 years of age) were performed.

ISGlobal is one of the world’s leading centres for the design, execution and evaluation of epidemiological studies, particularly randomized controlled trials of malaria intervention tools in children and pregnant women. It is associated with leading universities in Europe and offers longstanding postgraduate academic programmes, as well as training activities for health professionals and researchers in global health [31]. Together with international stakeholders, health organizations and academic institutions in LMIC, ISGlobal has coordinated and implemented capacity strengthening projects in Africa and Latin America. ISGlobal has also experience in the evaluation of capacity building programmes such as the WHO-TDR Clinical Research Development Fellowships Scheme [21].

JCF was created in 1991 by the Hospitallier Order of Saint John of God in Spain with the aim to fight poverty by creating and improving health and social care services in LMIC. It covers the areas of Cooperation, Humanitarian Aid, Voluntary Work and Awareness-raising and Education for Development. JCF and the SJCH started working together after the outbreak of the war in Liberia in 1990, driven by the needs of the country. In the last 25 years, both institutions have jointly managed more than 60 projects.

### Needs assessment

Between April 7 and 14, 2015, an ISGlobal resident doctor with microbiological background, provided technical advice on laboratory processes and infrastructure, biosecurity procedures and overall management of the laboratory for the reopening of the laboratory at the SJCH after the Ebola outbreak. The needs in infrastructure, personnel and training were evaluated through face-to-face interviews with staff from the SJCH and examination of procedures in the laboratory. Between May and June 2016, a research capacity needs assessment was carried out with the objective of understanding the organizational context of the SJCH, its resources, governance, and relationships with the communities, as well as the staff’s skills, motivations and capacities. The assessment also aimed at better informing the content, materials and timelines for the capacity building programme, to identify context constrains and possibilities, and to landscape locally-available facilitators, training venues and materials.

### Call for trainees, interviews and selection

Three groups of trainees were targeted for participation in the training programme: (i) SJCH clinical, laboratory, pharmaceutical, social work, and administrative staff; (ii) Academia and government key stakeholders in Liberia; (iii) Community leaders and other influential community representatives from Congo Town. An open call for applicants was launched in April 2016. The training programme was presented at a round of meetings held at the Liberia Medicines and Health Products Regulatory Authority (LMHRA), the University of Liberia-Pacific Institute for Research and Evaluation (UPL-PIRE), the Ministry of Health and Social Welfare (MoHSW) Emergency Operations Centre, the Mother Pattern College of Health Sciences (MPCHS), and the LIBR and the National Reference Laboratory. Community leaders were contacted during an exploratory visit to seven of the Congo Town communities. Group conversations were held at the communities’ *palava* with the community chiefs, chair ladies, and youth leaders.

SJCH and LMHRA applicants were interviewed face-to-face in May 2016. A structured interview guide was used to gather information about their professional background, experience in humanitarian aid and clinical research, knowledge on GCLP, motivation, and availability to participate in all the training stages. Information on IT-literacy and accessibility to computers was also collected to identify context constraints and possibilities (including connectivity, hardware, software). Candidates were shortlisted based on: (i) their background and motivation to receive training; (ii) impact of their participation on their routine clinical activities; and (iii) their potential role in research activities. LHMRA and MPCHS candidates were proposed by management staff of both institutions. Community leaders identified influential community members who were engaged in traditional decision-taking platforms, and able to mobilize the population in future health research.

### eLearning mentoring programme

A 5-month GCLP Mentoring Programme was created in the Moodle-enabled ISGlobal eLearning environment and conducted between August and December 2016 among SJCH staff. The eLearning programme aimed to facilitate the comprehension of the theoretical content offered during the training programme and further reflect on research ethics. The GCLP Mentoring Programme consisted of four modules that included theoretical content, scientific articles, links to external training resources (i.e., Global Health Training Centre’s webinars), exercises and assignments, and an in-built discussion forum and chat functionalities. A mentor (the SELeCT project manager) was available to provide support if required, via email or face-to-face in Monrovia. The requirements to participate in this programme were to have a University Diploma and/or College Certificate, and a signed motivation form.

### Workshops

A set of four workshops were conducted between August 2016 and June 2017. First, a 4-day presence-based, hands-on workshop on International Conference on Harmonization Guidelines for Good Clinical Practice (ICHE6 GCP) was conducted in August 2016. The objective was to help participants gain an understanding on the foundations of medical ethics, the different types of study designs that apply to health research, the GCP and GLP principles, and the role of community engagement in research. The content of the Workshop was informed by the ICH E6-GCP Handbook [32], the WHO-TDR GLP Guidelines [33], the UNAIDS Good Participatory Practice guidelines [34] and the Council for International Organizations of Medical Sciences Ethical Guidelines [35]. It included theoretical sessions, group discussions, educational videos, and practical exercises such as role plays (Additional file 1).

Scientific articles describing biomedical research conducted in Liberia were used as case studies to guide group work. Second, a 2-day workshop on research communication was conducted in September 2016 among community members. It included theoretical sessions on medical research ethics, types of health research, community mapping and mobilization. Group exercises aimed at identifying cultural and ethical issues to be considered when planning research and community sensitization in Liberia. Third, a 6-day workshop on Standard Operating Procedures (SOPs) was conducted between January 30 and February 3, 2017 (Additional file 2). It focused on the preparation of SOPs for Administration, Clinical, Community education, Laboratory, and Pharmacy Departments at the SJCH. The sessions started with the identification of documents needed for the specific department, the SOP format and the contents of each specific SOP. Finally, a 3-day workshop on scientific writing was conducted in June 2017 among SJCH staff, stakeholders and community members. The objective was to strengthen trainees’ capacities in the communication of research results and the preparation of a research article (Additional file 3). The workshop included eight theoretical sessions on analysis and presentation of research data, preparation and submission of a research article, including publication ethics. The workshop ended with a session on how to effectively disseminate research results to the communities. During six working group sessions, the participants practiced writing an article based on data obtained during the research project conducted at the SJCH.

### Research project

**Fig. 2:**
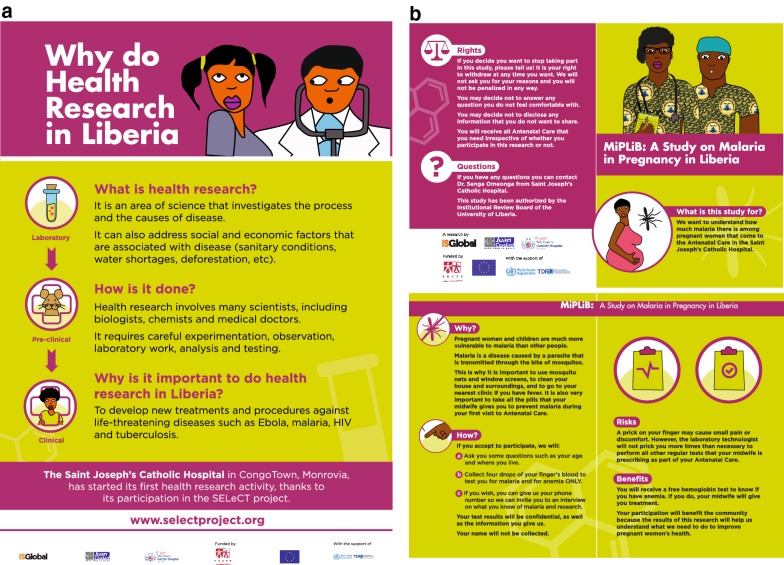
Poster (**a**) and Leaflet (**b**) describing the value of research and the malaria research project conducted among pregnant women at the Saint Joseph’s Catholic Hospital

The objectives of this practical exercise were to help SJCH trainees gain skills in the recruitment of participants in research, seeking for informed consent, collecting and managing data, handling, analysis and shipment of infectious specimens. A series of discussions with the SJCH management team were held in May 2016 to jointly define the research subject for the practical exercise. Protocols, consent documents, recruitment forms, refusal forms and sensitization materials (i.e. poster and brochures; Fig. 2) were developed between June and September 2016. The research protocol was approved by the UL-PIRE Institutional Review Board in Monrovia and by the Hospital Clínic Health Research Ethics Committee (Barcelona, Spain). Project-related activities were conducted between October 2016 and June 2017.

### Training assessment

The trainees were asked to complete a questionnaire to assess their knowledge on GCLP before the training programme. The questionnaire (Additional file 4) consisted in 32 items distributed in eight thematic blocks: informed consent; recruitment and retention; research protocol and responsibilities; research misconduct; confidentiality and privacy; participant safety and adverse events; institutional review board and subject protection; quality assurance and record-keeping. The same questionnaire with 13 extra questions was completed by trainees at the end of the training programme. Other measures of the effectiveness of the training programme were presentations by the trainees in international forums as well as published papers.

## Results

### Design of the training programme

**Fig. 3:**
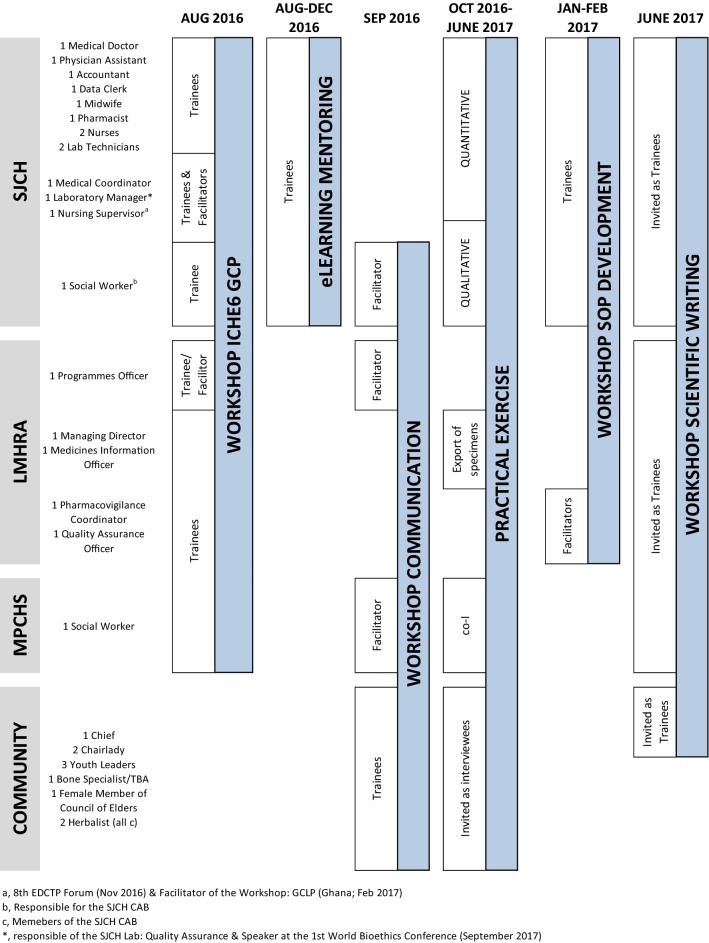
Activities of the training program

The first visit to the SJCH in 2015 identified several needs, including SOPs for laboratory processes and techniques, a data management system, community engagement activities, a system for calibration and monitoring of instruments, as well as an organizational plan of the laboratory personnel. The SJCH staff had varied levels of experience but there was no continuous education programme. Overall, they were proud of the hospital’s status previous to the Ebola outbreak, and the role of its laboratory as reference centre for the Liberian Health System, and were motivated by the idea of recovering these standards. Based on this assessment, the training programme pursued the building of capacities for conducting biomedical research and clinical trials through a hybrid approach consisting of presence-based workshops on GCP and GLP, SOP development and communication abilities, and a continuous eLearning platform with the follow-up of a mentor (Fig. 3).

### Training participants

By June 2016, 24 SJCH staff members, 11 (46%) of them female, applied to the training call. Their mean age was 38.4 years (standard deviation 8.7). Eight were staff from the Nursing Department. All were Liberian except three who were from Democratic Republic of Congo (DRC), one from Rwanda, and one from Cameroun. Twenty-two of the 24 candidates were individually interviewed. Only two candidates held a post-graduate degree in the biomedical field: a medical doctor and a pharmacist who had completed a MSc Epidemiology in Cuttington School (Liberia) and a post-graduate in Medical Biology at the University of Antwerp (Belgium), respectively. Six reported previous involvement in clinical research: a community-based survey to assess the prevalence of malaria, a clinical research on high blood pressure inhibitor drugs in Kinshasa (DRC), an oral HIV self-testing study in Ghana, and two clinical studies on antibiotic resistance and co-trimoxazole pharmacokinetics in HIV patients from Rwanda. In preparation for his MSc Finance thesis in Liberia, the SJCH accountant led a qualitative research to assess the impact of risk management in organizations. A laboratory technologist collected urine specimens to run parasite tests in a USAID-led survey in Liberia. Seven of the applicants had experience working as clinicians or as humanitarian aid workers in other SSA countries. The majority (n = 15) were engaged as SJCH staff during the 2014–2015 Ebola outbreak. Whilst some candidates (n = 12) had attended courses on research fundamentals and research ethics at college, no candidate had previously received GCLP training. The Nursing Director and Medical Coordinator had experience on the development of SOPs for eclampsia, antenatal care, diabetes mellitus, arterial hypertension and Ebola virus disease during the outbreak. Only four were able to describe what GCLP entitles. The others confounded GCLP with Clinical Practice Guidelines [36] and Infection Prevention and Control. Drawing on this confusion, many manifested that they were motivated to participate in the training to improve the healthcare they were providing to their patients (Table 1). Fourteen of the twenty-two candidates who were individually interviewed were finally invited as participants in the training programme. The majority were satisfied with the retribution offered by the SJCH. Only the midwives said they were partially satisfied. A physician assistant said that he was more interested in acquisition of knowledge than in compensation. A laboratory technician said “*I’m happy just to learn*!”.

**Table 1:** Excerpts from candidates at the interviews

From SJCH
I’d love to be part in this new thing that will benefit people in a positive way
Add knowledge and understand disease conditions that we have at St Joseph. Help us improve in the management of these conditions, and maybe even develop new medication or finding new ways to prevent them
Improve my knowledge to be better as a lab coordinator, so I’ll build my capacity as lab personnel and I’ll be able to contribute in data/medical discussions
I’ll get new ideas to help me improve my work and my staff’s skills
(I want to take on this training) for the country, for the community and for God
I want to advance my ability working for an iNGO. (I want to) improve my cv
From LMHRA
Capacity at LMHRA is lacking, there is no biostatistician, no health informatics and no epidemiologist
We have not really been involved in trials in Liberia. So this is going to be an added value. Many trials have closed down and only PREVAIL is ongoing

Following presentations at external institutions, the LMHRA and the MPCHS proposed four and one staff, respectively, as trainees. All LMHRA staff were registered male pharmacists with experience working with the MoHSW and other international organizations, and were actively engaged in the review of clinical trial protocols. The LMHRA expressed its interest in participating in the training since they wished to conduct similar capacity-building initiatives (Table 1). Some candidates expressed their interest in becoming training facilitators of sessions on SOP Development, Trial Site Preparedness, Pharmacovigilance, Safety Effects Reporting, and Trial Monitoring. The staff identified by the MPCHS was an associate professor for the BSc Social Work degree. The SJCH Social Work department identified ten community representatives who were invited to receive training in GCP and community mobilization for research.

### Training

A total of 18 staff members were selected for the Training programme: 14 from the SJCH, three from the LMHRA, and one from MPCHS (Table 2). Of these, four were invited to assist as co-facilitators: three from the SJCH (i.e. Medical Director, Nursing Director, Laboratory Manager) and one from the LMHRA (Programmes Officer). The *ICHE6 GCP Workshop* was accredited by the Transcellerate Biopharma Inc. Mutual Recognition Programme [37]. Topics included GCLP guidelines, ethical and legal regulation of biomedical research, essential documents for the conduct of a clinical trial, data collection and monitoring (Additional file 1). Based on the results of the pre-training questionnaire, emphasis was made on quality assurance, record-keeping, ethics regulation, and safety and adverse events reporting. All selected trainees attended the *ICHE6 GCLP Workshop* and received a certificate of completion. The workshops, eLearning-related activities and exercises were straightforward and in a language and formats the trainees were familiar with (e.g. ‘facebook’-like forums and webinars they could reproduce in private ‘youtube’ channels). Timelines to submit assignments were flexible to avoid disrupting local health care [38], and the facilitator’s mentoring support to the trainees was enhanced as much as possible and, whenever possible, face-to-face.

**Table 2:** Profile of shortlisted trainees

Role	SJCH	LMHRA	MPCHS	Congo Town
Trainees	1 Medical doctor	1 Medicine information officer		3 Youth leaders
	1 Pharmacist			1 Chief
	1 Physician assistant			2 Chairladies
	1 Accountant			1 Bone specialist
	1 Data clerk			2 Herbalists
	1 Midwife			1 Female member of the council of elders
	2 Nurses			
	2 Lab technicians			
Trainees and co-facilitators	1 Medical coordinator	1 Programmes officer	1 Social worker	
	1 Nursing supervisor	1 Pharmacovigilance coordinator		
	1 Laboratory manager			
	1 Social worker			
Total	14	3	1	10

*SJCH* Saint Joseph’s Catholic Hospital, *LMHRA* Liberia Medicines and Health Products Regulatory Authority, *MPCHS* Mother Pattern College of Health Sciences

The SJCH staff that participated in the *ICHE6 GCP Workshop* received credentials to log into the *eLearning* platform. Based on the input received during the interviews, the eLearning-related materials were created in formats the trainees were familiar with. Formats and timelines for their assignments were flexible. As trainees had no experience in writing scientific texts, they were rather asked to review and comment scientific articles and ethics documents. Since computer literacy and access to networked computers was limited, tablets were provided. By the end of the GCLP Mentoring Programme, the trainees had completed all modules as well as the online Clinical Research and GCP Courses offered by the Global Health Training Centre [39] and the National Drug Abuse Treatment-Clinical Trials Network [40].

The Communication workshop was facilitated, under the guidance of the SELeCT project manager, by the SJCH social worker, the MPCHS social worker, and the LMHRA programmes officer, who had attended the ICHE6 GCP. The ten community representatives that attended the workshop also manifested their interest in constituting the SJCH Community Advisory Board (CAB). They were summoned every second month to attend a CAB meeting chaired by the SJCH social worker. The aim was to build a platform to enhance community participation in healthy research activities.

SJCH trainees participated in the SOP Workshop. Four SJCH staff members were identified as training facilitators (Fig. 3). The Workshop led to the development of 5 sets of SOPs: Administration, Community education, Data management, Laboratory, and Pharmacy. The final workshop on Scientific Writing, facilitated by ISGlobal’s scientific writer and the SELeCT project manager, focussed on how to communicate research results and how to prepare a research article. One community chief, one chairlady and one youth leader, all CAB members, also participated in the workshop (Fig. 3). During the working group sessions, the attendants conducted a preliminary analysis of the demographic and laboratory data from the participants in the research project, discussed the study findings, and collaborated in writing a first draft of the manuscript reporting the main results.

### The research project

Group discussions with the SJCH management team identified malaria as one of the most prevalent infectious diseases in the hospital (Table 3). Given SJCH’s interest in maternal health, the practical exercise was framed on malaria in pregnancy. A protocol entitled ‘*The burden of malaria during pregnancy in Monrovia, Liberia, after the Ebola outbreak*’ (hereafter MiPLiB) was developed [41]. The research was designed as a mixed-methods cross-sectional study. The quantitative component aimed to assess the prevalence of *Plasmodium falciparum* infection among pregnant women attending a first antenatal care visit at the SJCH, through the collection of samples for molecular methodologies. The qualitative component aimed at exploring barriers and opportunities for pregnant women to accept participating in malaria research in Liberia.

**Table 3:** Top 10 diseases at the Saint Joseph’s Catholic Hospital

OPD morbidity				Causes of admissions			Causes of death		
No.	Condition	n	%	Condition	n	%	Condition	n	%
1	Pregnancy and related complications	5020	33.0	Labour	1228	38.2	Cardiopulmonary failure	67	17.2
2	Malaria	1953	12.8	Malaria	306	9.5	Respiratory distress	20	5.1
3	Gynaecological condition	951	6.2	Anemia	67	2.1	Anemia	15	3.8
4	Pneumonia	356	2.3	Gastroenteritis	46	1.4	Malaria	15	3.8
5	Skin diseases and ulcers	301	2.0	Hypertension	39	1.2	Hypertension	15	3.8
6	Hypertension	267	1.8	Gynaecological condition	33	1.0	Septicaemia	14	3.6
7	Diarrhoeal diseases	229	1.5	Hernia	32	1.0	Hypoglycemia	13	3.3
8	Other acute respiratory infections	157	1.0	Eclampsia	31	1.0	Aspiration pneumonia	8	2.1
9	Typhoid fever	129	0.8	Hypoglycemia	28	0.9	HIV/AIDS	8	2.1
10	Acute ear infection	22	0.1	Caesarean Section	23	0.7	Cardiorespiratory arrest	5	1.3
	All others	5834	38.3	All Others	1378	42.9	All others	210	53.8
	Total	15,219		Total	3211		Total	390	

From the Saint Joseph’s Catholic Hospital Annual Report 2015

With the hospital management’s advice, ten SJCH trainees were identified as playing a significant role during the practical exercise. Supporting staff from Nursing, Laboratory and Data Management Units at the SJCH were also involved in the practical exercise. The CAB helped to plan the research project taking into consideration local perceptions on biomedical research, as well as to prepare the materials and strategies to inform potential participants of the study. One trainee was tasked as co-principal investigator of MiPLiB and responsible of overseeing the study conduct. Three were responsible for the sampling and recruitment of pregnant women and for obtaining their informed consent. Three trainees were tasked to collect blood samples from the participants, and to handle and ship them to the ISGlobal laboratory in Barcelona as Category B (UN 3373) specimens, in accordance with International Air Transport Association instructions [42]. One trainee captured all demographics and laboratory data into the case report form manager software OpenClinica^®^. As trainees had no experience in qualitative research and social sciences research methodologies were not included in the training, the qualitative component was led by the SELeCT Project Manager. Two trainees from the SJCH manifested interest in qualitative methodologies and were engaged in the identification of key informants, the analysis and interpretation of the interviews, the presentation of findings at the CAB meetings, and the preparation of scientific articles.

The protocol was submitted to the Institutional Review Board of the UL-PIRE (Monrovia) and the Health Research Ethics Committee of the Hospital Clínic (Barcelona) in July 2016, and ethical clearance was obtained at the end of August 2016. Permission and sample transfer agreements were also obtained from the UL-PIRE and the SJCH, respectively. Recruitment of 195 study participants in the quantitative component [43] lasted from October 2016 until June 2017. Molecular analysis of samples was conducted at ISGlobal between July and September 2018. Writing of the quantitative study manuscript started during the Scientific Writing workshop (June 2017), with the final manuscript being submitted to peer-reviewed journals by January 2018.

### Training outcomes

Twelve (67%) out of the 18 trainees invited to participate in the Training Programme and 9 (90%) of the 10 community members satisfactorily completed their training tracks. The eighteen candidates filled in the pre-training questionnaire. Overall, female and male candidates responded correctly to 50% and 56% of the questionnaire’s items, respectively. The candidates scored better in the sets of questions pertaining to informed consent (66% correct answers); recruitment and retention (64%); research protocol and responsibilities (62%); and investigational new drugs and research misconduct (60%). Mean percentage of correct answers per participant was 53% (standard deviation 9.5%). Reasons for not completing the post-training questionnaire included two deaths, one migration out of Liberia, two dismissals from the SJCH and four disengagements from the course. Post-training questionnaires were completed by 9 of the 12 trainees who completed the training. Among them, 7 showed a positive increase in the percentage of correct answers (mean increase of 14%, standard deviation 8), while a decrease of 2% and 6% was observed in 2 of the trainees (Table 4).

**Table 4:** Knowledge change pre- and post-training among trainees from the Saint Joseph’s Catholic Hospital

Position	Age	Sex	Pre-GCLP (32 questions)		Post-GCLP (45 questions)		Δ (%)	Reason for not evaluation
			Score	%	Score	%		
Laboratory technologist	34	M	16	50	24	53	3	NA
Accountant	29	M	17	53	23	51	− 2	NA
Pediatrician	39	F	19	59	37	82	23	NA
OPD midwife	53	F	12	38	18	40	3	NA
Nurse	35	F	14	44	26	58	14	NA
Nursing supervisor	32	F	18	56	34	76	19	NA
Social worker	30	F	19	59	36	80	21	NA
Medical director	55	M	24	75	39	87	12	NA
Laboratory manager	39	M	24	75	31	69	− 6	NA
Physician assistant	50	M	18	56				Died in Jan 17
Project officer	55	M	18	56				Died in Jan 17
Data manager	33	M	19	59				Fired in April 17
Nurse	39	F	15	47				Migrated in April 17
Laboratory supervisor	48	M						Fired in March 17

The community members did not participate in the pre/post-training test

*Δ* difference between pre- and post-training scores, *NA* not applicable

**Fig. 4:**
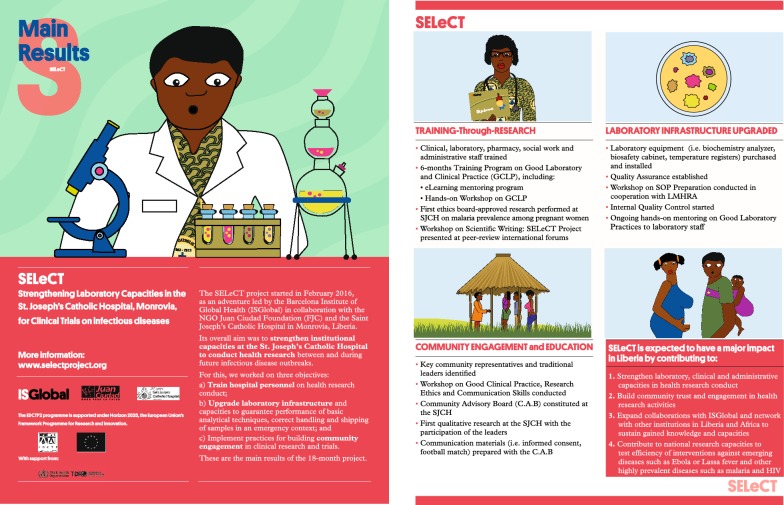
Brochures describing main achievements of the SELeCT project

The activities of the training programme and the practical exercise were communicated to stakeholders through brochures (Fig. 4), at the 8th EDCTP Forum (November 2016) in Lusaka (Zambia) [41] and at the American Society of Tropical Medicine and Hygiene Annual Meetings (November 2017 and 2018) [44, 45]. The Laboratory Director at the SJCH presented the project at the 1st World Bioethics Conference (September 2017) in El Escorial (Spain). Scientific outcomes were published in 3 peer-reviewed papers describing the prevalence of *P. falciparum* infection among pregnant women at first antenatal visit [43], the barriers and opportunities for Liberian pregnant women to participate in malaria research [46], and the community perceptions on the value of malaria research for pregnant women [44]. Finally, one of the trainees (Nurse Director) participated as a facilitator in a GCP workshop conducted at the St John of God Hospital in Koforidua (Ghana) in February 2017, and in the frame of a new EDCTP-funded LMHRA-led project that kicked off in mid-2017.

## Discussion

There is growing awareness worldwide of the importance of strengthening research capacity in LMICs to improve health, equity and development [47]. This perception has given rise to an interest in the impact achieved by different capacity building initiatives and the lessons that can inform future programmes. This report describes a research capacity-building initiative conducted in the Saint Joseph’s Catholic Hospital in Monrovia (Liberia) that combined training for healthcare professionals, regulatory authority members and community representatives, and the participation of trainees in a post-training research project.

Identifying existing and potential research capacities at the SJCH was a key first step in the project. A preliminary visit to the SJCH in April 2015 during the post-Ebola reopening of the hospital identified key areas for improvement, such as monitoring, documentation and planning for continuous education. The personal interviews conducted in June 2016 among SJCH applicants showed that the hospital staff lacked previous exposure to research, and training resources were adapted to provide the basic fundaments of health research. Whilst the layout of the hands-on GCLP Workshop cannot be modified because it needs to meet basic requirements [37], the content of the eLearning Platform was designed to take into account the trainees’ professional background as well as their role in the community or in their institutions. To enable an appropriate environment for research, the training programme was approached from a systemic perspective and also targeted administrative staff and stakeholders. The project also acknowledged the importance of engaging with the communities for the successful translation of research findings, and community members were invited to participate in the training programme. The practical research exercise included a social component through qualitative focus group discussions to characterize community perceptions on the aetiology, prevention and therapeutics of malaria, the utility of malaria research [48] to identify barriers and opportunities to participate in studies in Liberia [46].

The ten community representatives attended the workshop and constituted the SJCH Community Advisory Board (CAB). Although these community members were limited in their methodological skills, they were able to understand and follow the ethical principles for biomedical research and the main strategies to communicate the results of research projects. Their role was key for the qualitative inquiry conducted in mid-2016 with the aim of understanding the community members’ perceptions and attitudes towards research as well as on the contextual aspects that may deter or motivate pregnant women to participate in malaria research [46, 48]. The SJCH CAB members provided advice for the design of these qualitative studies. CAB members also provided insights on the appropriateness of the informed consent and data collection procedures used in a quantitative study aiming to assess the prevalence of malaria among pregnant women attending the SJCH [43]. Overall, the training of community representatives was key for the conduct of programme activities and the successful involvement of the community in the research.

The training programme faced several challenges. First, the eLearning mentoring programme was not easily adopted by the trainees. Some SJCH staff members did not have access to networked computers at work (e.g. the nurses and midwives), many lacked good computer literacy skills, and internet connection was erratic in Liberia. Although devices (i.e. tablets) were provided to facilitate access to the eLearning platform and the training facilitator was available to provide support in case of difficulties, the trainees found it difficult to follow the modules and exercises. This might be partly due to shortcomings of the Moodle platform interface design and to their daily workload in their consultations. Face-to-face trainings, individual communication and group discussions were more efficient to obtain fruitful insights into the topics studied. Second, the trainees did not have much experience in documentation procedures and quality assurance, and elaborating all these documents turned out to be an arduous process. Third, there is a paucity of research in Liberia and few local case studies were found to guide explanations and practical exercises in the workshops. Although examples from other studies were used, familiar examples (such as the Partnership for Research on Ebola Vaccines in Liberia) were more helpful for apprehending the fundaments of health research and medical ethics. This might no longer be a limitation for future training programmes in the country, given the myriad of studies that have been conducted as a result of the Ebola outbreak [49–51]. Finally, expectation of *per diems* was a cumbersome challenge [52]. Advancing professional careers by acquiring new knowledge, networking with international research institutions, and participating in international conferences was not attractive enough to some of the trainees who dropped out of the training programme. Decoupling training from per diem benefits could increase the chances of including in the training only health personnel genuinely interested in research. A public discussion on how this problem can impede achievement of operational objectives is urgently required if continuous and sustainable training in research is pursued.

This training programme had several limitations. First, a moderate rate of completion may have limited the success of the training programme and the performance of the practical exercise. This modest improvement may be explained by the relatively acceptable score that the trainees had before the training (out of 13, 10 of them gave correct answers for more than 50% of the questions, with 2 of them having a score of 75%). Moreover, we cannot discard the inadequacy of the pre- and post-training test to capture the knowledge gained during the training, which included not only theoretical concepts but also skills difficult to quantify in a questionnaire. Second, 18 months was a relatively short time to carry out the training activities, and the programme did not target important tools for biomedical research such as data management, monitoring and evaluation, computer-assisted statistics and qualitative data analysis software, which should be covered in future training activities. For this reason, ISGlobal took the lead in the writing of the research protocol, the statistical analysis and the qualitative data analysis. Third, this training programme was largely driven by funding from the global North, and lacked South-South collaborations that could foster regional partnerships. Building sustainable research capacity in SSA calls for locally led and run initiatives that draw on existing regional capacities and funds to ensure local ownership and active support.

In spite of being the first training-through-research project ever implemented at the SJCH, the trainees managed to participate in a mixed-methods research and prepare research outputs for international scientific meetings and peer-reviewed journals. Employing both qualitative and quantitative methods in this training programme allowed the trainees to get a more comprehensive picture of the structures, perceptions and processes of biomedical research. Regular meetings were also organized to discuss research prospects and progress outcomes of ongoing research. Moreover, trainees were encouraged to submit abstracts and apply for scholarships to attend regional and international conferences. Overall, this learn-by-doing experience was useful for the SJCH trainees to conduct simple, descriptive biomedical research studies. More sophisticated study designs, such as prospective cohort studies or randomized control trials, will require further training and investment in health information systems, laboratory infrastructure and site monitoring for compliance with ethics and GCLP standards [53].

In parallel to knowledge acquisition, the training programme emphasized building capacities in knowledge transfer. Some of the trainees were encouraged to contribute as co-trainers and to replicate the ICHE6 GCP workshop in other institutions (Koforidua Hospital in Ghana) and in the context of a new EDCTP-funded project that started in Monrovia in mid-2017. Capitalizing on these individuals’ research and education experience as well as on their leadership position within their respective organizations, can have a profound impact on the success of training activities by ensuring their sustainability and promoting transfer of acquired knowledge to other national institutions.

Evaluating the effectiveness of research capacity strengthening projects is crucial to improve ongoing initiatives, demonstrate their impact, and justify continued investment. However, there is limited research literature on validated methodologies to assess research capacities in low income-countries [54]. This is not due to a paucity in ongoing training activities [5, 55–58], but rather reflects the challenge of evaluating capacity building initiatives and the lack of harmonized metrics for evaluation. The impact of the training programme conducted at the SJCH was assessed through quantitative indicators such as the number of people trained, the acquired knowledge measured through pre- and post-questionnaires and the number of publications in peer-reviewed journals. By the end of the training programme, a 67% (12/18) of the trainees and 90% (9/10) of the community representatives completed their respective training tracks. This modest completion of the training programme highlights the chronic problem of high staff turnover and brain-drain which impede sustaining a critical mass for biomedical research. Strategic planning to engage trained individuals in research activities, provide opportunities for research staff to disseminate their findings in scientific fora, and promote translation of new knowledge to policy and practice might motivate them to stay in their workplaces. Continuous research education of the SJCH staff through appropriate funding mechanisms and with the collaboration of health authorities and academia is necessary to ensure the sustainability of the gains obtained though this initiative.

## Conclusions

The training programme conducted at the SJCH was the first training-through-research experience that the SJCH had ever engaged in. This experience was successful in building capacities to conduct a descriptive biomedical research on malaria, in involving the community, in fostering interest in carrying out health and social research studies, and in the training-of-trainers. The practical research exercise on malaria during pregnancy, the first actual research implemented by the trainees as co-investigators, provided data on the burden of *P. falciparum* in pregnancy which can be useful to plan future control and preventive tools for malaria. The challenge is to ensure long-term commitments that give the trainees and the SJCH the means to remain active in research. Establishing indicators to measure the effectiveness of research capacity-building initiatives would strengthen the case for investing in such efforts.

## Additional files

**Additional file 1.** Program of the GCLP Workshop.

**Additional file 2.** Program of the SOP Workshop.

**Additional file 3.** Program of the Workshop in scientific writing.

**Additional file 4.** Pre- and post-training questionnaire.

## Abbreviations

CAB: Community Advisory Board
DRC: Democratic Republic of Congo
EDCTP: European and Developing Countries Clinical Trial Partnership
GCLP: Good Clinical Laboratory Practice
HRC: Health research capacities
ICHE6 GCP: International Conference on Harmonization Guidelines for Good Clinical Practice
ISGlobal: Barcelona Institute for Global Health
JCF: Juan Ciudad Foundation
LIBR: Liberian Institute for Biomedical Research
LMHRA: Liberia Medicines and Health Products Regulatory Authority
LMIC: Low- and middle-income countries
MiPLiB: The burden of malaria during pregnancy in Monrovia, Liberia, after the Ebola outbreak
MoHSW: Ministry of Health and Social Welfare
MPCHS: Mother Pattern College of Health Sciences
SELeCT: Strengthening laboratory capacities in the St. Joseph’s Catholic Hospital, Monrovia, for clinical trials on infectious diseases
SJCH: Saint Joseph’s Catholic Hospital
SOP: Standard Operating Procedures
SSA: Sub-Saharan Africa
UPL-PIRE: University of Liberia-Pacific Institute for Research and Evaluation
WHO-TDR: World Health Organization Special Programme for Research and Training in Tropical Diseases
