# Supplementary material for: Training through malaria research: building capacity in good clinical and laboratory practice in Liberia
Source: Malar J. 2019 Apr 17;18:136. doi: 10.1186/s12936-019-2767-1 (PMC6471755; doi:10.1186/s12936-019-2767-1)
Supplement: Supplementary file 1 — Additional file 1. Program of the GCLP Workshop. [file 12936_2019_2767_MOESM1_ESM.docx]

**Additional file 1.** Program of the GCLP Workshop.

|  | **August 22-26, 2016** | | | | |
| --- | --- | --- | --- | --- | --- |
|  | **Monday** | **Tuesday** | **Wednesday** | **Thursday** | **Friday** |
| **9h** | SELeCT Project | Study Protocol  Investigators Brochure  Essential documents | Informed Consent of Trial Subjects | Clinical Trial Protocol: Trial objectives, design, selection and withdrawal of subjects, assessment of efficacy and safety, data handling and record keeping, ethics | Introduction to World Health Organization Special Programme for Research and Training in Tropical Diseases Good Laboratory Practice Standards |
| **10h** | Introduction to Biomedical Research | Definition of Investigator, Sub-investigator & Sponsor Investigator  Responsibilities (Safety Reporting) | Records and Reports  Progress Reports  Safety Reporting  Final Report |  | GLP: History, Objectives, Resources, Rules |
| **11h** | **Tea break** | **Tea break** | **Tea break** | **Tea break** | **Tea break** |
| **11.30h** | ICH GCP Overview (Definition & Objectives)  Principles of ICH GCP | Adequate Resources  Medical Care of Trial Subjects  Compliance with Protocol | Premature Termination or Suspension of a Trials  Sponsor (Quality Assurance and Quality Control; CRO; Medical expertise) | Community Engagement in Biomedical research | GLP: Characterization, Documentation, Results, Quality Assurance |
| **13.30h** | **Lunch break** | **Lunch break** | **Lunch break** | **Lunch break** | **Lunch break** |
| **14.30h** | IEC/IRB (Composition, Protocols, Procedures and Amendment(s)) | Investigational Products  Randomization Procedures and Unblinding | Sponsor (Monitoring, Audit, Adverse Drug reaction Reporting) | Good Participation Practices | Post-test questionnaire  Closing Remarks |
| **16h** | Open Discussion, Q&A, Review | Open Discussion, Q&A, Review | Open Discussion, Q&A, Review | Open Discussion, Q&A, Review |  |
