# Supplementary material for: Training through malaria research: building capacity in good clinical and laboratory practice in Liberia
Source: Malar J. 2019 Apr 17;18:136. doi: 10.1186/s12936-019-2767-1 (PMC6471755; doi:10.1186/s12936-019-2767-1)
Supplement: Supplementary file 2 — Additional file 2. Program of the SOP Workshop. [file 12936_2019_2767_MOESM2_ESM.docx]

**Additional file 2.** Program of the SOP Workshop.

|  | **January 23-28, 2016** | | |
| --- | --- | --- | --- |
|  | **Monday** | **Tuesday** | **Wednesday** |
| **9h-16h** | Administration and development | Clinical research | Data management |
|  | **Thursday** | **Friday** | **Saturday** |
| **9h-16h** | Laboratory research | Pharmacy | Community education and involvement |
