# Supplementary material for: Training through malaria research: building capacity in good clinical and laboratory practice in Liberia
Source: Malar J. 2019 Apr 17;18:136. doi: 10.1186/s12936-019-2767-1 (PMC6471755; doi:10.1186/s12936-019-2767-1)
Supplement: Supplementary file 3 — Additional file 3. Program of the Workshop in scientific writing. [file 12936_2019_2767_MOESM3_ESM.docx]

**Additional file 3.** Program of the Workshop in scientific writing.

|  | **June 15-17, 2017** | | |
| --- | --- | --- | --- |
|  | **THURSDAY** | **FRIDAY** | **SATURDAY** |
| **9h** | Communication of research results:  - Oral form (Lecture, short presentations)  - Written form (Abstract/poster for congress, Original research article) | How to prepare a research article:  - Common terms used in health studies  - Limitations, bias, recommendations  - Conclusions and acknowledgements | Publication ethics:  - Authorship, Plagiarism  - Funding  - Funding and conflict of interests |
| **9.30h** | How to prepare a research article:  - Structure  - Access to scientific literature (Pubmed, etc) and reference management | **Working Group III**  MipLib Prevalence Article preparation: Introduction, Methods, Results  (Continued) | **Working Group V**  MipLib Prevalence Article preparation (Discussion)  (Continued) |
| **10h** | **Working Group I**  MipLib Prevalence Article preparation: Audience, Structure, Baseline information |  |  |
| **11h** | **Breakfast break** | | |
| **11.15h** | How to prepare a research article:  - Analysis & Data presentation | Implication for Operations  - FGD on MiPLiB Qualitative Findings | General tips for disseminating effectively to the communities. Defining the audience, the message and the means |
| **11.45h** | **Working Group II**  MipLib Prevalence Article preparation: Introduction, Methods, Results | **Working Group IV**  MipLib Prevalence Article preparation: Discussion | **Working Group VI**  Group presentation of exercise |
| **13.30h** |  |  | Submission of manuscripts |
| **14h** | **LUNCH BREAK** | | |
