# Supplementary material for: Training through malaria research: building capacity in good clinical and laboratory practice in Liberia
Source: Malar J. 2019 Apr 17;18:136. doi: 10.1186/s12936-019-2767-1 (PMC6471755; doi:10.1186/s12936-019-2767-1)
Supplement: Supplementary file 4 — Additional file 4. Pre- and post-training questionnaire. [file 12936_2019_2767_MOESM4_ESM.docx]

**Additional file 4.** Pre- and post-training questionnaire.

# A. INSTITUTIONAL REVIEW BOARD and RESEARCH SUBJECTS PROTECTION

1. **May a clinical study begin enrolling participants before Institutional Review Board approval is granted?**
2. Yes, if the Ministry of Health has authorized the study.
3. Yes, if potential participants are willing to be enrolled as soon as possible.
4. Never.
5. Institutional Review Board approval is not necessary to conduct clinical trials.
6. **What information should be provided to an IRB for review at the initiation of a study? Choose the best response:**
7. Only an advertisement to recruit participants that included how much they will be paid.
8. The informed consent form only.
9. The study protocol (and amendments), the information to be given to the participants (informed consent, advertisements), the Investigator Brochure (or drug label), any other relevant safety information, and an outline of the qualifications of the Investigator.
10. The study budget and two Letters of Support from the study site District of Health.
11. **The three key principles that underlie the current system of human research protections (respect for persons, beneficence, and justice) were established in which of the following:**
12. The Belmont Report.
13. Declaration of Human Rights.
14. Geneva Convention.
15. Holy Bible.
16. **The purpose of an IRB is to safeguard the rights, safety, and well-being of all human research participants regardless of their ethnic affiliation, age, sexual orientation, religion or socio-economic status. This statement is:**
17. TRUE.
18. FALSE.
19. **Choose the best response. Vulnerable populations that may be coerced into participating in research are:**
20. Pregnant women, prisoners, children, mentally disabled persons.
21. Pregnant women, priests, politicians, children.
22. Adult men, fishermen, mine workers.
23. Hospital personnel, members of the armed forces.
24. Answers A and D are correct.

# B. INFORMED CONSENT

1. **The consent document must state reasonably foreseeable risks or inconveniences to the participants and, when applicable, to an embryo, fetus, or nursing infant:**
2. Always.
3. It is not necessary to disclose foreseeable risks to an embryo.
4. Only if the participant is literate and educated.
5. The consent document must state the risks but not the inconveniences.
6. **As part of the consent process, each participant must be informed that she/he has a right to withdraw only at the beginning of a study.**
7. FALSE
8. TRUE
9. **As part of the consent process, participants must be informed if there is any payment for participating in the study:**
10. Yes, if the payment is 10,00$/day and above.
11. It is not necessary when the payment consists in a grocery voucher.
12. Yes, this information must be part of the informed consent process.
13. Information on payment for participation must be given by a research nurse once informed consent has been provided by the participant.
14. **Is the following statement true? All informed consent documents should be written in non-technical language that potential participants would understand; rather, the language should be consistent with the proposed participants educational level, cultural views, and familiarity with research.**
15. Yes, the statement in true.
16. No, the statement is false.
17. **When a research involves pregnant women as participants, both mother and father must be informed about any risk of the research on the fetus. However, the father’s consent is not required if…:**
18. The father’s identify cannot be determined.
19. The pregnancy resulted from rape.
20. The father has other wives.
21. A and B are true.
22. The father works for the pharmaceutical industry.

# C. CONFIDENTIALITY AND PRIVACY

1. **All of the following is participants’ protected health information (PHI) that must be kept confidential except:**
2. E-Mail address.
3. Cellphone number.
4. Latest blood pressure.
5. Name and surname
6. Passport Number.
7. **Medical and research records of any person that participates in an alcohol and drug abuse program, are subject to disclosure and confidentiality requirements.**
8. TRUE
9. FALSE
10. **Choose the best response. Confidential information about a study participant may be disclosed to local public health authorities when then participant has… :**
11. Yellow fever, cholera, Ebola.
12. Malaria, influenza, HIV-infection.
13. HIV-infection, malaria, diabetes.
14. Diabetes, hallux valgus, pancreatitis.
15. **Which one of the following practices you would not recommend for maintaining the confidentiality of research participants?**
16. Limit access to all data that identifies participants.
17. Store paper records in locked cabinets.
18. Assign security codes to computerized records.
19. Send all data via Dropbox to the Study Sponsor for her/him to store the participants’ data safely.
20. Remove face sheets that contain identifiers, such as names and addresses.
21. **De-identifying individually identifiable health information involves…:**
22. Removing vital signs and laboratory examinations from all study records.
23. Removing all personal identifiers and all identifiable health information from the study records.
24. Keeping personal health information but updating the computer’s firewall to keep the data confidential.
25. Eliminating both participants’, sponsors’ and investigators’ personal health information from the study records.

# D. PARTICIPANT SAFETY AND ADVERSE EVENTS

1. **Who should be responsible for the accurate documentation, investigation, and follow-up of all safety reports and responsible to ensure that local Institutional Review Boards involved in a study are fully informed or any safety issues that may arise from the study protocol?**
2. Sponsor
3. Research nurse
4. Lead investigator
5. Research assistant
6. Data and safety manager
7. **An Adverse Event may be defined as any untoward medical occurrence in a research assistant or lead investigator that gets administered a pharmaceutical product and that does not necessarily have a causal relationship with this treatment:**
8. TRUE
9. FALSE.
10. **A HIV-infected study participant takes a study medication and, the next week, reports suffering from herpes zoster. Is this an Adverse Event?**
11. Yes, it is.
12. No, it is not.
13. **An Adverse Drug Reaction is:**
14. A predictable effect, associated with the use of a drug that always occurs as part of the pharmacological action of the drug.
15. An undesirable effect, reasonably associated with the use of a drug, that may occur as part of the pharmacological action of the drug or may be unpredictable in its occurrence.
16. An effect associated with the use of an investigational drug by a study participant once the study has been concluded.
17. An undesirable and predictable Adverse Event that has been communicated to the study participant during the informed consent process.
18. **Regarding the goal of a Data and Safety Monitoring which of the following is INCORRECT:**
19. Ensure that risks of participation in a clinical study are minimized as far as is reasonably possible.
20. Keep a record of study finances, including per diem and retribution received by the participants.
21. Ensure the integrity of the data collected in a clinical study.
22. Stop a study if safety concerns arise.

# E. QUALITY ASSURANCE and RECORD-KEEPING

1. **Who is responsible for Quality Assurance?**
2. Institutional Review Board auditors.
3. Sponsor.
4. All members of the study protocol team.
5. Only the Principal Investigator.
6. The Quality Assurance Manager.
7. **On-site and remote monitoring is required and may occur at any given study site:**
8. Before a trial begins.
9. Whilst it is in progress
10. After a study concludes.
11. All of the above.
12. **One of the main purposes of monitoring is:**
13. Collect data for dissemination of study outcomes at international conferences.
14. Verify that trial data are accurate and that statistical analysis is done as per protocol specifications.
15. Verify that the rights and well-being of participants are protected.
16. Report to the Department of Health on trial progress and occurrence of adverse events.
17. **Essential study documents, such as protocol and informed consents, may be audited or inspects by Quality Assurance Monitors. This statement is:**
18. False, only the Sponsor can audit essential study documents.
19. False, only the Principal Investigator can audit essential study documents.
20. False, only an independent an external auditing company can audit essential study documents.
21. This statement is true.
22. This is true, provided the study involves seeking for informed consent from study participants.
23. **Which one of the following is NOT an Essential Document?**
24. Signed study protocol.
25. Investigator’s Brochure.
26. Copyright transfer of study outcomes to peer-review journals.
27. Instructions for handling and dispensing investigational drugs.
28. Documentation of study personnel’s qualifications.

# F. INVESTIGATIONAL NEW DRUGS and RESEARCH MISCONDUCT

1. **An investigator participating in a clinical trial is responsible for protecting the rights, the safety and welfare of trial participants. Part of this responsibility is:**
2. Ensuring all subjects are consented appropriately and documented using the IRB-approved Informed Consent form.
3. Ensuring that poor uneducated women are enrolled so they may benefit from the compensation to the participants that the study provides.
4. Enrolling subjects into the study who meet the exclusion criteria.
5. Ensuring that the names of all subjects participating in the study are available to the general public.
6. **Which one of the following statements is FALSE?**
7. Neither an investigator nor a sponsor may advertise an investigational new drug as safe or effective for the investigational purpose.
8. Neither an investigator nor a sponsor may distribute commercially an investigational new drug.
9. Neither an investigator nor a sponsor may stop an investigation after finding good evidence that the investigational new drug is safe and effective.
10. Neither an investigator nor a sponsor may charge for an investigational new drug in a clinical trial unless the drug is being provided for recreational use.
11. **Choose the best response. Clinical trials of an investigational new drug are generally conducted in…:**
12. Four phases. The fourth one involving healthy volunteers.
13. Three phases. The second one involving several thousand participants.
14. Four phases. The fourth one after the drug has been approved for marketing.
15. Three phases. The third one being conducted at a laboratory and involving healthy female rats.
16. **Choose the best response. Research misconduct is:**
17. Fabrication or making up data or results and recording or reporting them.
18. A, C and E are true.
19. Falsification or changing research materials, equipment or processes or altering or omitting data or results.
20. Differences of opinion between the Research Team and the Principal Investigator that lead to verbal abuse.
21. Plagiarism or using another person’s ideas, processes, results or words without giving appropriate credit.
22. **Which of the following activities may be viewed as research misconduct?**
23. Refusing to enroll men older than 45 years old in a trial on investigational new drugs for painful dysmenorrhea.
24. Refusing to enroll a potential participant that meets all inclusion criteria because he discloses that he is a HIV-infected male sex worker.
25. Dispute over who must be placed as First Author in an article to be submitted to a peer-review journal.
26. Keeping informed consents in a locked cabinet to prevent unauthorized data clerks from accessing them.
27. Reporting on use of grant funds two weeks after deadline given by grant managers.

# G. RESEARCH PROTOCOL and RESPONSIBILITIES

1. **Which section of a randomized trial protocol should contain a description of the measures for de-identification of data gathered in focus group discussions?**
2. Focus Group Discussions are never conducted in randomized trials.
3. This information is not relevant and must not be reported in the protocol.
4. Data Management Section.
5. Statistics and Data Analysis.
6. **A protocol violation occurs whenever a study staff person performs any action that does not adhere to the research protocol and may consist in omissions, additions, and/or changes in any procedure described in the protocol. This statement is:**
7. TRUE
8. FALSE
9. **A written description of a change(s) to some aspect of the study as described in the research protocol is a…:**
10. Appendix to a Research Protocol
11. Research Modification
12. Updated Research SOPs
13. Research Amendment.
14. Nothing of the above.
15. **All study participants should receive appropriate medical care for both study-related adverse events as well as all medical conditions unrelated to study participation.**
16. TRUE
17. FALSE
18. **Primary care physicians should be informed that a participant has got infected with malaria parasites during a trial, if…:**
19. The participant has a primary care physician.
20. The participant agrees that her/his primary care physician may be informed.
21. A and B are correct.
22. A qualified physician affiliated with the study must treat the participant. Hence, there is no need to notify the participant’s primary care physician.
23. Primary care physicians shall never be informed about any participant’s involvement in a clinical trial.

# H. RECRUITMENT AND RETENTION

1. **When deciding to select some people for a study and exclude others, investigators must ensure that participants are chosen for reasons that are directly related to the problem being studied and not simply because of their availability, their compromised position, or their vulnerability. This statement is:**
2. TRUE
3. FALSE
4. **Should women and minorities be adequately represented in the study population of pharmaceutical trials?**
5. Yes, so the research findings will be meaningful for these groups and so that members of these groups can share in the benefits of the research.
6. Not necessarily. Women are already overrepresented in clinical research.
7. No, minorities are vulnerable populations and need be protected and excluded from participation in research involving investigational new drugs.
8. It depends. Sexual minorities must be adequately represented whilst there is no need to ensure adequate representation of tribal or ethnic minorities.
9. **Choose the WRONG answer. Elements of successful recruitment strategy include:**
10. Identify barriers for recruitment.
11. Choose appropriate staff members to conduct recruitment.
12. Increase monetary incentives to potential participants.
13. Develop a recruitment plan during the protocol planning stage.
14. Avoid specifying unnecessarily restrictive exclusion criteria in the protocol.
15. **Can researchers use web sites to recruit and screen participants?**
16. Yes
17. No
18. **To successfully retain women participants in a trial, researchers may try to:**
19. Phone women’s husbands to ask them to convince their partners not to discontinue their participation in the trial.
20. Treat participants with respect and being considerate of their expressed barriers to attend trial consultations.
21. Top up the monetary incentives to participate in the trial with beauty salon vouchers.
22. Warn participants in their first visit to the trial site that they may not withdraw from the study.

# I. GOOD LABORATORY PRACTICES

1. **Choose the best response. One of the aims of the Good Laboratory Practice is to:**
2. Make the incidence of false negatives more obvious.
3. Make the incidence of false positives more obvious.
4. Make the incidence of false positives less obvious.
5. A and B are correct.
6. **Choose the best response. One of the principles of the Good Laboratory Practice is to:**
7. Help scientists obtain results that are reliable, repeatable, and auditable.
8. Help scientists obtain results that are realistic, unique, and publishable.
9. Help scientists obtain results that are accepted, acceptable and accepting of local values.
10. Help scientists obtain results that are replicable, realistic and reciprocal.
11. **Record of equipment suitability, calibration, checking and maintenance demonstrate that the laboratory SOPs have been followed and that the equipment used in any study is adequate for the job and performing to its specification. Does this statement agree with Good Laboratory Practice:**
12. Yes, it does.
13. No, it does not.
14. **Choose the WRONG answer. According to Good Laboratory Practice, a typical animal house should have separations maintained by provision of areas for:**
15. Different studies.
16. Quarantine.
17. Storage of materials.
18. Staff qualifications.
19. Necropsy.
20. **Choose the WRONG answer. According to Good Laboratory Practice:**
21. A procedure should ensure that Curriculum Vitae(s) exist for all personnel in a standard approved format.
22. The content of Curriculum Vitae(s) should be defined in SOPs and verified regularly in QA audits.
23. The organization chart may form part of a quality manual describing the nature of the institution.
24. A Master Schedule is the record of planned staff allocation to laboratory premises.

Questions 2,9, 12, 20, 24, 27, 33, 39 and 41-45 were only included in the post-training questionnaire.

**RESPONSES**: 1. C; 2. C; 3. A; 4. A; 5. E; 6. A; 7. A; 8. C; 9. A; 10. D; 11. C; 12. A; 13. A; 14. D; 15. B; 16. C; 17. B; 18. A; 19. B; 20. B; 21. C; 22. D; 23. C; 24. D; 25. C; 26. A; 27. C; 28. C; 29. B; 30. B; 31. C; 32. A; 33. D; 34. A; 35. C; 36. A; 37. A; 38. C; 39. A; 40. B; 41. D; 42. A; 43. A; 44. D; 45. D.
